# A Tribute to Two Master Teachers of Immunology

**DOI:** 10.3390/biomedicines11082178

**Published:** 2023-08-02

**Authors:** Roberto Paganelli

**Affiliations:** Internal Medicine, Saint Camillus International University of Medical and Health Sciences, 00131 Rome, Italy; roberto.paganelli@unicamillus.org

A Special Issue dedicated in memory of Prof. Fernando Aiuti is a special tribute to a clinician who led the field of Clinical Immunology in Italy and introduced the entire Italian medical and academic scene to it. Therefore, I wish to thank all the Guest Co-Editors of this Special Issue for undertaking and supervising this effort. Fernando excelled in the study of patients with primary immunodeficiencies (PIDs) who are the veritable experiments of nature (in the words of R. A. Good [1,2]), teaching us important lessons on immunology.

I was a young postgraduate student when Professor Good—then at the Memorial Sloan-Kettering Cancer Center in New York—visited our Clinic in Rome, gave an impressive seminar on the new horizons that studies of PID patients were opening, and hinted to the vast unknown fields of research appearing in front of us. I started my research career in the field with enthusiasm with the group of Prof. Aiuti, under the supervision of only slightly older colleagues (Giuseppe Luzi, Raffaele D’Amelio, Franco Pandolfi) who gave me the rudiments of the art, but Robert Good has always remained my compass in the journey. Dr. Patrizia Ammirati from our group even joined the Sloan-Kettering Cancer Center, and I visited her on my first trip to the United States in 1982. But before that, in 1978, I left Rome following Prof. Aiuti’s decision to open up new areas of study through specific research stages abroad, and joined the Immunology Department of the Institute of Child Health in London under the supervision of Professor J. F. Soothill, a world-class expert in PIDs and in food allergies. There, I conducted projects under the direct supervision of Prof. Soothill, Prof. M. W. Turner and Dr. A. R. Hayward, but then Dr. Roland J. Levinsky became my Ph.D. tutor for the following three years. Already in 1979 we published our first results in *The Lancet* [3]. He was to become the co-author of the first genetic breakthrough in the field of PIDs, with the discovery of mutation of the *btk* gene as the cause of X-linked agammaglobulinemia (Bruton type) [4]. I am indebted to both John and Roland for what they taught me during my four years in London, and for their help at the very beginning of my career in research.

The only possible circumstance for me to have this inspiring start was the choice of Professor Fernando Aiuti as my mentor and guide since 1975, when I joined his Lab at La Sapienza University in Rome, Italy. His openness and will to disseminate knowledge in immunology—he held crowded courses to young and less young scientists coming from all over the country, and attracted the attention of the media and the Academy to the benefit of the discipline—were so revolutionary at the time; he also had a sense for the research fields which would lead to future advances, as in the case of human immunodeficiency virus epidemics, which he embraced with enthusiasm and with assiduous presence in the media to deliver correct scientific information to the public. He led many initiatives for the dissemination of medical and scientific knowledge concerning immunology. I was in charge as Secretary, to organize a first meeting of European scientists/physicians who were active in the study and care of patients with primary immunodeficiency disorders, back in the spring of 1983 in Rome. I found a name for it—the European Group of Immune Deficiencies or EGID—and the two-day meeting was held at the Serono Symposia headquarters with a very small budget but enthusiastic participation from Sweden, France, the UK, and even from Israel. One of the participants was the future Nobel prize winner Prof. Luc Montagnier, and his young coworker J. C. Chermann who presented unpublished data (submitted to Science— [5]) showing a viral particle in a case of lymphoadenopathy syndrome, the name given to symptoms preceding overt manifestations of AIDS. The EGID had a second meeting in Viterbo, organized by Prof. M. Fiorilli, and then a third one in Versailles, where it officially became the European Society for Immuno Deficiencies (ESID), which is still thriving [6,7,8,9,10,11]. I served on the Steering Committee for the European Registry, which was an original idea of Prof. Aiuti, who started the Italian Registry [12] which then became the backbone of the IPINet, the Italian network for immunodeficiencies [13]. Soon after that, Fernando funded an association for the care of patients with HIV infection, also promoting fundamental and clinical research into its cure and prevention, AnLAids. It is with this organization that the name of Professor Aiuti became known widely in Italy and abroad and associated with HIV and AIDS. In previous years, he was often presumed to be a pediatrician for his studies on PIDs, where in fact he was always supported by Prof. Luisa Businco, a lifelong associate and excellent pediatrician who co-authored most of the seminal studies in this field [14,15,16,17]. This manuscript is therefore a tribute to my several Master Teachers. I shall spend now a few paragraphs highlighting the fundamental contribution made by Prof. Good to the study of immunology.

Robert Alan Good made major contributions to the understanding of several immunodeficiency diseases, describing for the first time eight of them; he contributed greatly to the recognition of the critical role of the thymus in developmental immunology. In 1968, Dr. Good performed the first successful allogeneic bone marrow transplantation on a child affected by the otherwise lethal condition of X-linked severe combined immunodeficiency [18], paving the way for cellular correction of genetically determined inborn errors of immunity, which Prof. Aiuti’s team followed a decade later [19,20], among a handful of other European centers [21]. Robert Good was the author of 2136 scientific publications [22], and in 1978 he was recognized as the most cited author over a 10-year period. Many of his students and coworkers, distinguished scientists and physicians, have published obituaries [22,23,24,25,26,27,28], remembering Robert Good as a giant, a genius, the father of modern immunology [24,27,29,30]. I wish to quote from Dr. Good’s description of his encounter with the patient affected by the syndrome [31] which has been named after him:

“Another experiment of nature that I encountered in 1952 proved enlightening. My surgical colleague Richard Varco asked me to see a farmer who was hospitalized for removal of a tremendous mediastinal mass. The appearance of this tumor had marked the onset of recurrent infections, including 16 bouts of pneumonia in eight years. The tumor was associated with a broadly-based immunodeficiency syndrome evidenced by profound hypogammaglobulinemia, deficiency of antibody production, deficiencies of all cell-mediated immunities, and low lymphocyte and plasma cell counts. Removal of the 540 g benign thymoma did not improve the immunodeficiency syndrome. This patient led us to ponder on the role played in immune responses by the thymus, which had long been discounted as a sort of vestigial organ.”

(Excerpt from ref. [1]).

It is then with gratitude that I wish to honor both Prof. Good and Prof. Aiuti for growing an entire generation of clinical immunologists, and the Co-Editors of this Special Issue are to be counted as among the best. My contribution to the Special Issue [32] is a tribute to two great teachers.

## References

[1] Good R.A. (1991). Experiments of nature in the development of modern immunology. Immunol. Today.

[2] Good R.A. (1968). Experiments of nature in immunobiology. N. Engl. J. Med..

[3] Paganelli R., Levinsky R.J., Brostoff J., Wraith D.G. (1979). Immune complexes containing food proteins in normal and atopic subjects after oral challenge and effect of sodium cromoglycate on antigen absorption. Lancet.

[4] Vetrie D., Vorechovský I., Sideras P., Holland J., Davies A., Flinter F., Hammarström L., Kinnon C., Levinsky R., Bobrow M. (1993). The gene involved in X-linked agammaglobulinaemia is a member of the src family of protein-tyrosine kinases. Nature.

[5] Barré-Sinoussi F., Chermann J.C., Rey F., Nugeyre M.T., Chamaret S., Gruest J., Dauguet C., Axler-Blin C., Vézinet-Brun F., Rouzioux C. (1983). Isolation of a T-lymphotropic retrovirus from a patient at risk for acquired immune deficiency syndrome (AIDS). Science.

[6] Conley M.E., Notarangelo L.D., Etzioni A. (1999). Diagnostic criteria for primary immunodeficiencies. Representing PAGID (Pan-American Group for Immunodeficiency) and ESID (European Society for Immunodeficiencies). Clin. Immunol..

[7] Guzman D., Veit D., Knerr V., Kindle G., Gathmann B., Eades-Perner A.M., Grimbacher B. (2007). The ESID Online Database network. Bioinformatics.

[8] De Vries E., Clinical Working Party of the European Society for Immunodeficiencies (ESID) (2006). Patient-centred screening for primary immunodeficiency: A multi-stage diagnostic protocol designed for non-immunologists. Clin. Exp. Immunol..

[9] Sewell W.A., Khan S., Doré P.C. (2006). Early indicators of immunodeficiency in adults and children: Protocols for screening for primary immunological defects. Clin. Exp. Immunol..

[10] Schatorjé E., van der Flier M., Seppänen M., Browning M., Morsheimer M., Henriet S., Neves J.F., Vinh D.C., Alsina L., Grumach A. (2016). Primary immunodeficiency associated with chromosomal aberration—An ESID survey. Orphanet J. Rare Dis..

[11] Pergent M., Haerynck F., Hoste L., Gardulf A. (2023). COVID-19 vaccination in patients with primary immunodeficiencies: An international survey on patient vaccine hesitancy and self-reported adverse events. Front. Immunol..

[12] Luzi G., Businco L., Aiuti F. (1983). Primary immunodeficiency syndromes in Italy: A report of the national register in children and adults. J. Clin. Immunol..

[13] Lougaris V., Pession A., Baronio M., Soresina A., Rondelli R., Gazzurelli L., Benvenuto A., Martino S., Gattorno M., Biondi A. (2020). The Italian Registry for Primary Immunodeficiencies (Italian Primary Immunodeficiency Network; IPINet): Twenty Years of Experience (1999–2019). J. Clin. Immunol..

[14] Businco L., Rubaltelli F.F., Paganelli R., Galli E., Ensoli B., Betti P., Aiuti F. (1986). Results in two infants with the DiGeorge syndrome-effects of long-term TP5. Clin. Immunol. Immunopathol..

[15] Aiuti F., Businco L., Fiorilli M., Galli E., Quinti I., Rossi P., Seminara R., Goldstein G. (1983). Thymopoietin pentapeptide treatment of primary immunodeficiencies. Lancet.

[16] Aiuti F., Businco L., Fiorilli M., De Martino M., Vierucci A. (1979). Fetal liver transplantation in two infants with severe combined immunodeficiency. Transplant. Proc..

[17] Businco L., Rezza E., Giunchi G., Aiuti F. (1975). Thymus transplantation. Reconstitution of cellular immunity in a four-year-old patient with T-cell deficiency. Clin. Exp. Immunol..

[18] Gatti R.A., Meuwissen H.J., Allen H.D., Hong R., Good R.A. (1968). Immunological reconstitution of sex-linked lymphopenic immunological deficiency. Lancet.

[19] Businco L., Rossi P., Paganelli R., Seminara R., Aiuti F. (1983). Immunologic reconstitution with bone marrow transplantation and thymic hormones in two patients with severe pure T-cell defects. Birth Defects Orig. Artic. Ser..

[20] Businco L., Rossi P., Paganelli R., Galli E., DiGilio G., Lulli P., Aiuti F. (1984). Bone marrow transplantation and thymopoietin pentapeptide treatment in two infants with immunodeficiency with predominant T-cell defects. Clin. Immunol. Immunopathol..

[21] Fischer A., Griscelli C., Friedrich W., Kubanek B., Levinsky R., Morgan G., Vossen J., Wagemaker G., Landais P. (1986). Bone-marrow transplantation for immunodeficiencies and osteopetrosis: European survey, 1968–1985. Lancet.

[22] Stiehm E.R. (2007). Robert A Good and Ludwig van Beethoven: Immortal beloveds: Summary of the Robert A Good Immunology Society: Perspective in Immunology 2006 June 9–12 2006 St. Pete Beach, Florida. Immunol. Res..

[23] Rose D.W. (2007). Robert A. Good, the March of Dimes, and immunodeficiency: An historical perspective. Immunol. Res..

[24] Ribatti D. (2006). The fundamental contribution of Robert A. Good to the discovery of the crucial role of thymus in mammalian immunity. Immunology.

[25] Pickett D., Day-Good N. (2008). Robert A. Good: Physician, scholar, scientist, teacher vignettes. Immunol. Res..

[26] O’Reilly R.J. (2003). Robert Alan Good, MD, PhD. Biol. Blood Marrow Transplant..

[27] Nezelof C., Seemayer T.A. (2004). Robert Alan Good ou les intuitions géniales d’un immunologiste [Robert Alan Good or the genius intuitions of an immunologist]. Rev. Prat..

[28] Modell F. (2007). Immunology today and new discoveries: Building upon legacies of Dr. Robert A. Good. Immunol. Res..

[29] Cooper M.D. (2003). In memoriam. Robert A. Good May 21, 1922–June 13, 2003. J. Immunol..

[30] Bahna S.L. (2015). Father of modern immunology-Robert A. Good *(*1922–2003). Ann. Allergy Asthma Immunol..

[31] Good R.A., Varco R.L. (1955). A clinical and experimental study of agammaglobulinemia. J. Lancet.

[32] Paganelli R., Di Lizia M., D’Urbano M., Gatta A., Paganelli A., Amerio P., Parronchi P. (2023). Insights from a Case of Good’s Syndrome (Immunodeficiency with Thymoma). Biomedicines.

